# Second-order topological insulators and loop-nodal semimetals in Transition Metal Dichalcogenides XTe_2_ (X = Mo, W)

**DOI:** 10.1038/s41598-019-41746-5

**Published:** 2019-03-27

**Authors:** Motohiko Ezawa

**Affiliations:** Department of Applied Physics, University of Tokyo, Hongo, 7-3-1, 113-8656 Japan

## Abstract

Transition metal dichalcogenides XTe_2_ (X = Mo, W) have been shown to be second-order topological insulators based on first-principles calculations, while topological hinge states have been shown to emerge based on the associated tight-binding model. The model is equivalent to the one constructed from a loop-nodal semimetal by adding mass terms and spin-orbit interactions. We propose to study a chiral-symmetric model obtained from the original Hamiltonian by simplifying it but keeping almost identical band structures and topological hinge states. A merit is that we are able to derive various analytic formulas because of chiral symmetry, which enables us to reveal basic topological properties of transition metal dichalcogenides. We find a linked loop structure where a higher linking number (even 8) is realized. We construct second-order topological semimetals and two-dimensional second-order topological insulators based on this model. It is interesting that topological phase transitions occur without gap closing between a topological insulator, a topological crystalline insulator and a second-order topological insulator. We propose to characterize them by symmetry detectors discriminating whether the symmetry is preserved or not. They differentiate topological phases although the symmetry indicators yield identical values to them. We also show that topological hinge states are controllable by the direction of magnetization. When the magnetization points the *z* direction, the hinges states shift, while they are gapped when it points the in-plane direction. Accordingly, the quantized conductance is switched by controlling the magnetization direction. Our results will be a basis of future topological devices based on transition metal dichalcogenides.

## Introduction

Higher-order topological insulators (HOTIs) are generalization of topological insulators (TIs). In the second-order topological insulators (SOTIs), for instance, topological corner states emerge though edge states do not in two dimensions, while topological hinge states emerge though surface states do not in three dimensions^1–15^. The emergence of these modes is protected by symmetries and topological invariants of the bulk. Hence, an insulator so far considered to be trivial due to the lack of the topological boundary states can actually be a HOTI. Indeed, phosphorene is theoretically shown to be a two-dimensional (2D) SOTI^16^. A three-dimensional (3D) SOTI is experimentally realized in rhombohedral bismuth^17^, where topological quantum chemistry is used for the material prediction^18^. Transition metal dichalcogenides XTe_2_ (X = Mo, W) are also theoretically shown to be 3D SOTIs^19,20^.

The tight-binding model for transition metal dichalcogenides has already been proposed, which is closely related to a type of loop-nodal semimetals^20^. A loop-nodal semimetal is a semimetal whose Fermi surfaces form loop nodes^21–25^. Especially, the Hopf semimetal is a kind of loop-nodal semimetal whose Fermi surfaces are linked and characterized by a nontrivial Hopf number^26–30^. There is another type of loop nodal-semimetals characterized by the monopole charge^21^. An intriguing feature is that loop nodes at the zero-energy and another energy form a linked-loop structure. The proposed model^20^ may be obtained by adding certain mass terms to this type of loop-nodal semimetals.

It is intriguing that topological boundary states can be controllable externally. Magnetization is an efficient way to do so. Famous examples are surface states of 3D magnetic TIs^31–34^, where the gap opens for out-of-plane magnetization, while the Dirac cone shifts for in-plane magnetization. Similar phenomena also occur in 2D TIs, which can be used as a giant magnetic resistor^35^. Recently, a topological switch between a SOTI and a topological crystalline insulator (TCI) was proposed^36^, where the emergence of topological corner states is controlled by magnetization direction. We ask if a similar magnetic control works in transition metal dichalcogenides.

In this paper, we investigate a chiral-symmetric limit of the original model^20^ constructed in such a way that the simplified model has almost identical band structures and topological hinge states as the original one. Alternatively, we may consider that the original model is a small perturbation of the chiral symmetric model. A great merit is that we are able to derive various analytic formulas because of chiral symmetry, which enable us to reveal basic topological properties of transition metal dichalcogenides. We find that a linking structure with a higher linking number is realized in the 3D model. We also study 2D SOTIs and 3D second-order topological semimetals (SOTSMs) based on this model. Depending on the way to introduce mass parameters there are three phases, i.e., TIs, TCIs and SOTIs in the 2D model. We find that topological phase transitions occur between these phases without band gap closing. Hence, the transition cannot be described by the change of the symmetry indicators. We propose symmetry detectors discriminating whether the symmetry is preserved or not. They can differentiate these three topological phases. Furthermore, we show that the topological hinge states in the SOTIs are controlled by magnetization. When the magnetization direction is out of plane, the topological hinge states only shift. On the other hand, when the magnetization direction is in plane, the gap opens in the topological hinge states.

## Result

### Hamiltonians

**Figure 1:**
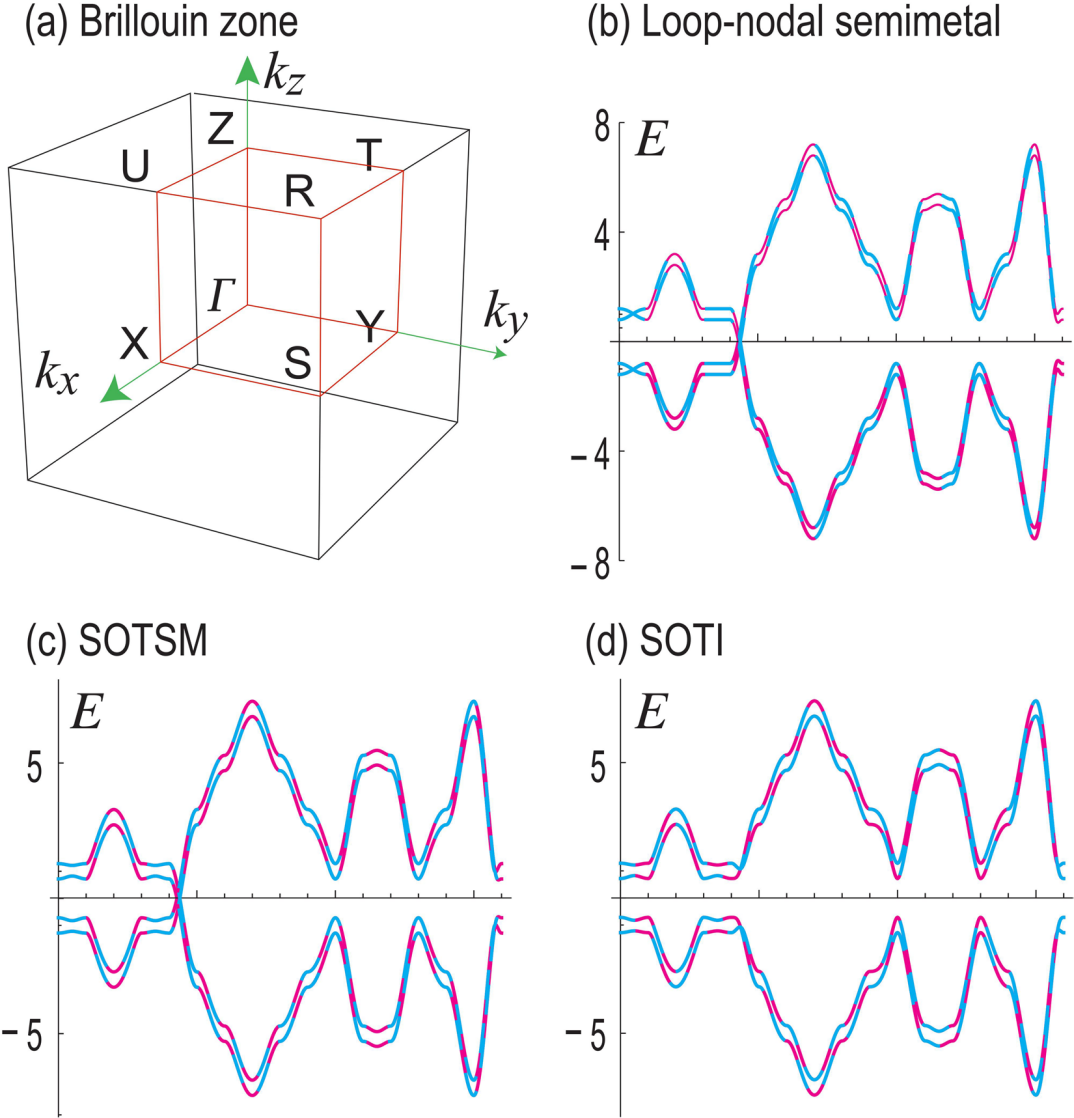
(**a**) Brillouin zone and high symmetry points. (**b**–**d**) Bulk band structures along the Γ-X-S-Y-Γ-Z-U-R-T-Z-Y-T-U-X-S-R-Γ line (**b**) for loop-nodal semimetal, (**c**) for SOTSM and (**d**) for SOTI. There are four bands in each phase. The dashed magenta curves represent the band structure of the chiral-symmetric model, while the dashed cyan curves represent that of the chiral-nonsymmetric model. They are indistinguishable in these figures. We have chosen *t*_*x*_ = *t*_*y*_ = 1, *t*_*z*_ = 2, *λ*_*x*_ = *λ*_*y*_ = 1, *λ*_*z*_ = 1.2, *m* = −3, *m*_2_ = 0.3, *m*_3_ = 0.2, *m*_*mv*1_ = −0.4, *m*_*mv*2_ = 0.2, *m*_Loop_ = 0.3 and *m*_SOTSM_ = 0.3.

Motivated by the model Hamiltonian^20^ which describes the topological properties of transition metal dichalcogenides *β*-(1T′-)MoTe_2_ and *γ*-(Td-)XTe_2_ (X = Mo, W), we propose to study a simplified model Hamiltonian,

1 $${H}_{{\rm{SOTI}}}={H}_{0}+{H}_{{\rm{SO}}}+{V}_{{\rm{Loop}}}+{V}_{{\rm{SOTSM}}},$$

with

2 $$\begin{array}{ccc}{H}_{0} & = & [m+{\sum }_{i=x,y,z}{t}_{i}\,\cos \,{k}_{i}]{\tau }_{z}\\  &  & +\,{\lambda }_{x}\,\sin \,{k}_{x}{\tau }_{x}+{\lambda }_{y}\,\sin \,{k}_{y}{\tau }_{y}{\mu }_{y},\end{array}$$

3 $${H}_{{\rm{SO}}}={\lambda }_{z}\,\sin \,{k}_{z}{\tau }_{y}{\mu }_{z}{\sigma }_{z},$$

4 $${V}_{{\rm{Loop}}}={m}_{{\rm{Loop}}}{\tau }_{z}{\mu }_{z},\,{V}_{{\rm{SOTSM}}}={m}_{{\rm{SOTSM}}}{\mu }_{x},$$

where *σ*, *τ* and *μ* are Pauli matrices representing spin and two orbital degrees of freedom. It contains three mass parameters, *m*, *m*_Loop_ and *m*_SOTSM_. The role of the term *m*_Loop_ is to make the system a loop-nodal semimetal, and that of the term *m*_SOTSM_ is to make the system a SOTSM. The Brillouin zone and high symmetry points are shown in Fig. 1(a). Although the band structure of the transition metal dichalcogenides is chiral nonsymmetric, the topological nature is well described by the above simple tight-binding model.

The original Hamiltonian contains two extra mass parameters and given by

5 $${H}_{{\rm{SOTI}}}^{^{\prime} }={H}_{0}+{H}_{{\rm{SO}}}+{V}_{{\rm{Loop}}}^{^{\prime} }+{V}_{{\rm{SOTSM}}}^{^{\prime} }$$

with

6 $${V}_{{\rm{Loop}}}^{^{\prime} }={m}_{2}{\tau }_{z}{\mu }_{x}+{m}_{3}{\tau }_{z}{\mu }_{z},$$

7 $${V}_{{\rm{SOTSM}}}^{^{\prime} }={m}_{mv1}{\mu }_{z}+{m}_{mv2}{\mu }_{x}\mathrm{.}$$

**Figure 2:**
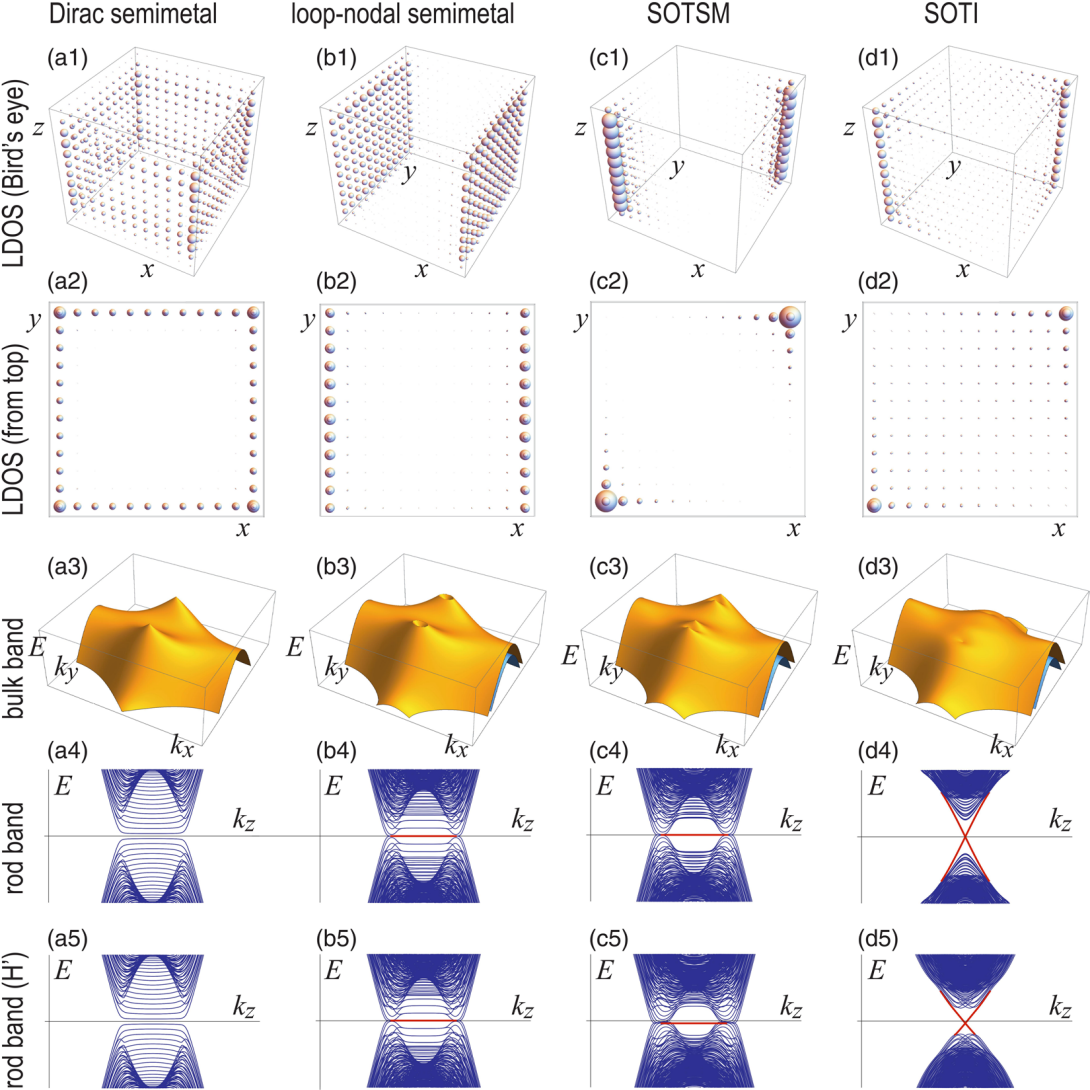
(**a1**–**d1**) Bird’s eye’s views of the LDOS of the zero-energy states: (**a1**) for *H*_0_ with surface zero-energy states on the four side surfaces; (**b1**) for *H*_Loop_ with surface zero-energy states on the two side surfaces; (**c1**) *H*_SOTSM_ with hinge-arc states at two pillars; (**d1**) *H*_SOTI_ with hinge states at two pillars. (**a2**–**d2**) Top view of the LDOS corresponding to (**a1**–**d1**). (**a3**–**d3**) Bulk band structures of valence bands along *k*_*x*_ = 0 plane for these Hamiltonians. (**a4**–**d4**) Band structures of the square rod along *z* direction for these Hamiltonians. (**a5**–**d5**) Corresponding rod band structures for the chiral-nonsymmetric Hamiltonian *H*′. In these two sets of figures red curves represent topological boundary states. The Parameters are the same as in Fig. 1.

The simplified model *H*_SOTI_ captures essential band structures of the original model ${H}_{{\rm{SOTI}}}^{^{\prime} }$. Indeed, the bulk band structures are almost identical, as seen in Fig. 1(b–d). The rod band structures are also very similar, as seen in Fig. 2(a4–d4,a5–d5), where the bulk band parts are found almost identical while the boundary states (depicted in red) are slightly different. Moreover, the both models have almost identical hinge states, demonstrating that they describe SOTIs inherent to transition metal dichalcogenides XTe_2_.

A merit of the simplified model is the chiral symmetry, {*H*_SOTI_(*k*_*x*_, *k*_*y*_, *k*_*z*_), *C*} = 0, which is absent in the original model, $\{{H}_{{\rm{SOTI}}}^{^{\prime} }({k}_{x},{k}_{y},{k}_{z}),C\}\ne 0$. Accordingly, the band structure of *H* is symmetric with respect to the Fermi level. Moreover, the bulk band structure is analytically solved. Here, the chiral symmetry operator is *C* = *τ*_*y*_*μ*_*z*_*σ*_*x*_ or *C* = *τ*_*y*_*μ*_*z*_*σ*_*y*_. Let us call the original model a chiral-nonsymmetric model and the simplified model a chiral-symmetric model.

The common properties of the two Hamiltonians *H*_SOTI_ and ${H}_{{\rm{SOTI}}}^{^{\prime} }$ read as follows. First, they have inversion symmetry *P* = *τ*_*z*_ and time-reversal symmetry *T* = *iτ*_*z*_*σ*_*y*_*K* with *K* the complex conjugation operator. Inversion symmetry *P* acts on *H*_SOTI_ as *P*^−1^*H*_SOTI_(***k***)*P* = *H*_SOTI_(−***k***), while time-reversal symmetry *T* acts as *T*^−1^*H*_SOTI_(***k***)*T* = *H*_SOTI_(−***k***). Accordingly, the Hamiltonian has the *PT* symmetry (*PT*)^−1^*H*_SOTI_(***k***)*PT* = *H*_SOTI_(***k***), which implies that H^*^ = *H*. Second, the *z*-component of the spin is a good quantum number *σ*_*z*_ = *s*_*z*_. Since we may decompose the Hamiltonian into two sectors,

8 $${H}_{{\rm{SOTI}}}={H}_{{\rm{SOTI}}}^{\uparrow }\oplus {H}_{{\rm{SOTI}}}^{\downarrow },$$

it is enough to diagonalize the 4 × 4 Hamiltonians. All these relations hold also for ${H}_{{\rm{SOTI}}}^{^{\prime} }$. The relation (8) resembles the one that the Kane-Mele model is decomposed into the up-spin and down-spin Haldane models on the honeycomb lattice^37–39^.

A convenient way to reveal topological boundary states is to plot the local density of states (LDOS) at zero energy. First, we show the LDOS for the Hamiltonian *H*_0_ in Fig. 2(a1). It describes a Dirac semimetal, whose topological surfaces appear on the four side surfaces. Then, we show the LDOS for the Hamiltonian

9 $${H}_{{\rm{Loop}}}={H}_{0}+{V}_{{\rm{Loop}}}$$

in Fig. 2(b1), where the topological surface states appear only on the two side surfaces parallel to the *y*-*z* plane. We will soon see that a loop-nodal semimetal is realized in *H*_Loop_. Next, we show the LDOS for the Hamiltonian

10 $${H}_{{\rm{SOTSM}}}={H}_{0}+{V}_{{\rm{Loop}}}+{V}_{{\rm{SOTSM}}}$$

in Fig. 2(c1), where a SOTSM is realized with two topological hinge-arcs. Finally, by including *H*_SO_, we show the LDOS for the Hamiltonian *H*_SOTI_ in Fig. 2(d1), where a SOTI is realized with topological two-hinge state.

### Topological phase diagram

The chiral-symmetric Hamiltonian *H*_SOTI_ is analytically diagonalizable. The energy dispersion is given by

11 $$E=\pm \sqrt{F\pm \sqrt{G}}$$

with

12 $$\begin{array}{ccc}F & = & {M}^{2}+{m}_{{\rm{Loop}}}^{2}+{m}_{{\rm{SOTSM}}}^{2}\\  &  & +\,{\lambda }_{x}^{2}{\sin }^{2}{k}_{x}+{\lambda }_{y}^{2}{\sin }^{2}{k}_{y}+{\lambda }_{z}^{2}{\sin }^{2}{k}_{z},\end{array}$$

13 $$G={({m}_{{\rm{SOTSM}}}{\lambda }_{x}\sin {k}_{x}-{m}_{{\rm{Loop}}}{\lambda }_{y}\sin {k}_{y})}^{2}$$

14 $$+\,2{M}^{2}({m}_{{\rm{Loop}}}^{2}+{m}_{{\rm{SOTSM}}}^{2}),$$

and

15 $$M=m+{\sum }_{i=x,y,z}{t}_{i}\,\cos \,{k}_{i}\mathrm{.}$$

The topological phase diagram is determined by the energy spectra at the eight high-symmetry points Γ = (0, 0, 0), *S* = (*π*, *π*, 0), *X* = (*π*, 0, 0), *Y* = (0, *π*, 0), *Z* = (0, 0, *π*), *R* = (*π*, *π*, *π*), *U* = (*π*, 0, *π*) and *T* = (0, *π*, *π*) with respect to time-reversal inversion symmetry. The energies at these high-symmetry points (*k*_*x*_, *k*_*y*_, *k*_*z*_) are analytically given by

16 $$E({k}_{i})={\eta }_{a}M({k}_{i})+{\eta }_{b}\sqrt{{m}_{{\rm{Loop}}}^{2}+{m}_{{\rm{SOTSM}}}^{2}},$$

where *η*_*a*_ = ±1 and *η*_*b*_ = ±1. The phase boundaries are given by solving the zero-energy condition (*E* = 0),

17 $${(m+{\eta }_{x}{t}_{x}+{\eta }_{y}{t}_{y}+{\eta }_{z}{t}_{z})}^{2}={m}_{{\rm{Loop}}}^{2}+{m}_{{\rm{SOTSM}}}^{2},$$

where *η*_*x*_ = ±1, *η*_*y*_ = ±1 and *η*_*z*_ = ±1. There are 16 critical points apart from degeneracy. When *t*_*x*_ = *t*_*y*_, the critical points are reduced to be 12 since *E*(*X*) = *E*(*Y*) and *E*(*U*) = *E*(*T*). Hence, solving *E* = 0 for *t*_*z*_, there are 6 solutions for *t*_*z*_ > 0, which we set as *t*_*n*_, *n* = 1, 2, 3, …, 6 with *t*_*i*_ < *t*_*i*+1_.

### Loop-nodal semimetals

We first study the loop nodal phase described by the Hamiltonian *H*_Loop_. The energy spectrum is simply given by

18 $$E=\pm \,\sqrt{{\lambda }_{x}^{2}{\sin }^{2}{k}_{x}+{(\sqrt{{\lambda }_{y}^{2}{\sin }^{2}{k}_{y}+{M}^{2}}\pm |{m}_{{\rm{Loop}}}|)}^{2}}.$$

The loop-nodal Fermi surface is obtained by solving *E*(***k***) = 0. It follows that *k*_*x*_ = 0 and

19 $${\lambda }_{y}^{2}{\sin }^{2}{k}_{y}+{M}^{2}(\mathrm{0,}\,{k}_{y},{k}_{z})={m}_{{\rm{Loop}}}^{2}.$$

**Figure 3:**
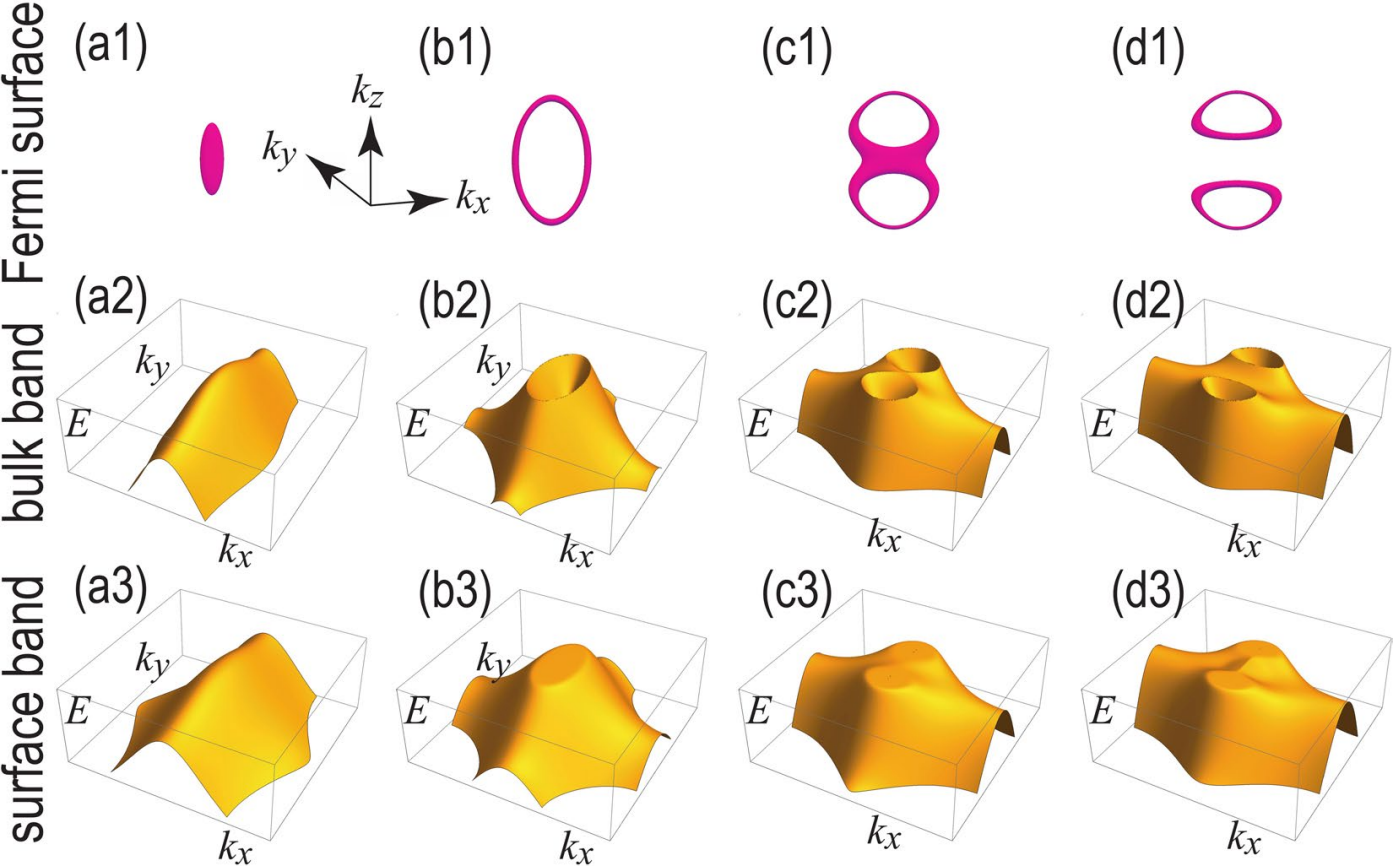
(**a1**–**d1**) Loop-nodal zero-energy Fermi surfaces for (**a1**) *t*_*z*_ = *t*_1_, (**b1**) *t*_1_ < *t*_*z*_ < *t*_2_, (**c1**) *t*_*z*_ = *t*_2_ and (**d1**) *t*_1_ < *t*_*z*_ < *t*_2_. (**a2**–**d2**) Band structures along *k*_*x*_ = 0 plane. (**a3**–**d3**) Drum-head surface states of the valence band along the *y*-*z* plane. *t*_*x*_ = *t*_*y*_ = 1, *λ*_*x*_ = *λ*_*y*_ = 1; *m* = −3, *m*_Loop_ = 0.75. In (**a2**–**d3**), only the valence bands are shown for clarity.

Loop nodes at zero energy exist in the *k*_*x*_ = 0 plane. They are protected by the mirror symmetry *M*_*x*_ = *τ*_*z*_*μ*_*z*_*σ*_*x*_ with respect to the *k*_*x*_ = 0 plane and the *PT* symmetry^21,40^. We show the band structure along the *k*_*x*_ = 0 plane in Fig. 3(a2–d2). We see clearly that the loop node structures are formed at the Fermi energy in Fig. 3(b2–d2). These loop nodes are also observed as the drum-head surface states, which are partial flat bands surrounded by the loop nodes as shown in Fig. 3(b3–d3). The low energy 2 × 2 Hamiltonian is given by

20 $$H=(\sqrt{{\lambda }_{y}^{2}{\sin }^{2}{k}_{y}+{M}^{2}}\pm |{m}_{{\rm{Loop}}}|){\sigma }_{z}+{\lambda }_{x}\,\sin \,{k}_{x}{\sigma }_{x},$$

where *σ* is the Pauli matrix for the reduced two bands.

In addition, there are loop nodes on the *k*_*y*_ = 0 plane at *E* = −*m*_Loop_, which are determined by

21 $${\lambda }_{x}^{2}{\sin }^{2}{k}_{x}+{(M({k}_{x},\mathrm{0,}{k}_{z})-{m}_{{\rm{Loop}}})}^{2}={m}_{{\rm{Loop}}}^{2}.$$

**Figure 4:**
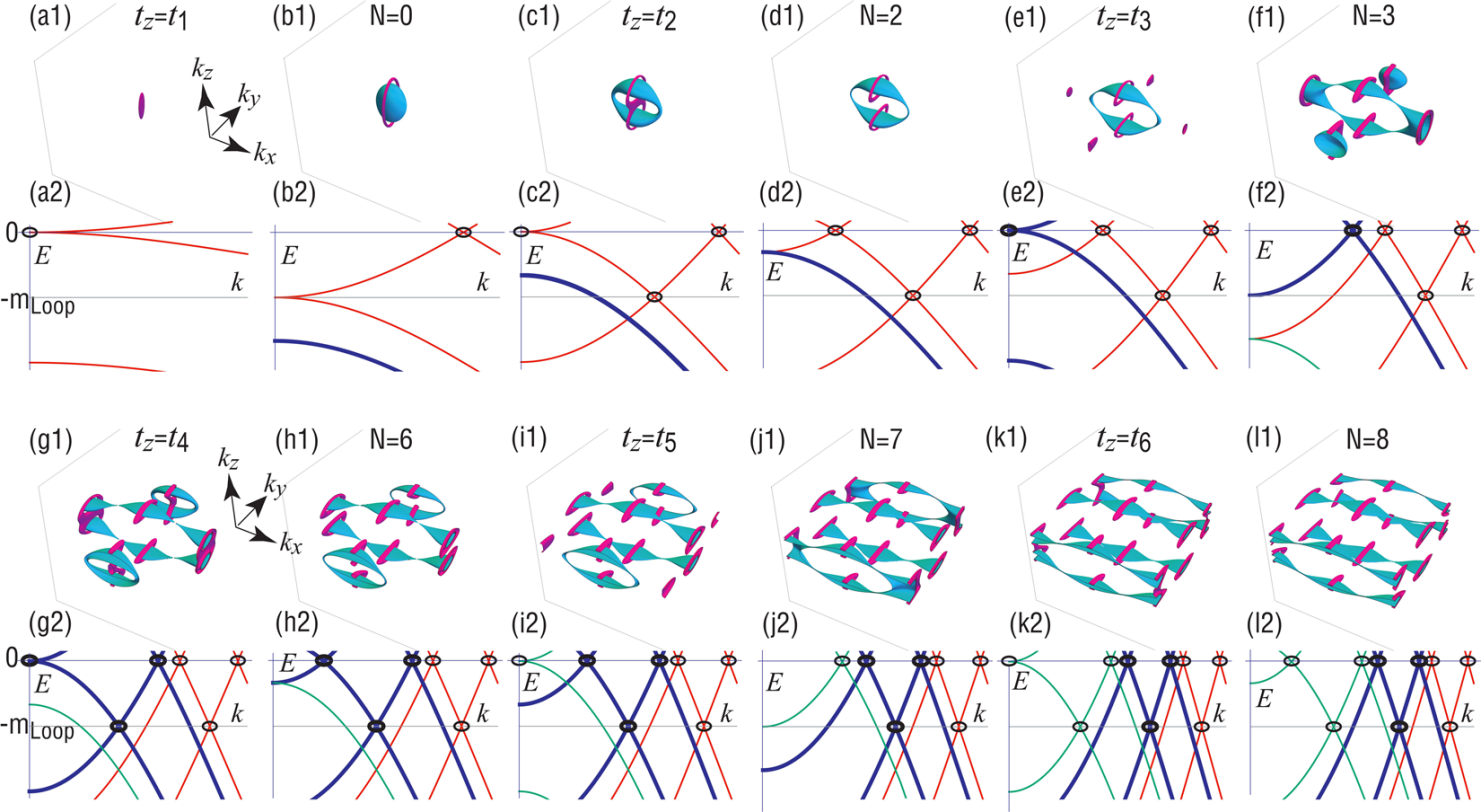
Evolution of linking structures for various *t*_*z*_. (**a**) *t*_*z*_ = *t*_1_, (**b**) *t*_1_ < *t*_*z*_ < *t*_2_, (**c**) *t*_*z*_ = *t*_2_, (**d**) *t*_2_ < *t*_*z*_ < *t*_3_, (**e**) *t*_*z*_ = *t*_3_, (**f**) *t*_3_ < *t*_*z*_ < *t*_4_, (**g**) *t*_*z*_ = *t*_4_, (**h**) *t*_4_ < *t*_*z*_ < *t*_5_, (**i**) *t*_*z*_ = *t*_5_, (**j**) *t*_5_ < *t*_*z*_ < *t*_6_, (**k**) *t*_*z*_ = *t*_6_ and (l) *t*_*z*_ > *t*_6_. (**a1**–**l1**) Loop-nodal Fermi surfaces at the zero-energy (magenta) and at *E* = −*m*_Loop_ (cyan). They are linked, whose linking number *N* is shown in figures. (**a2**–**l2**) Band structure along the Γ-*Z* line (red), the *X*-*U* and *Y*-*T* lines (thick blue curves representing double degeneracy) and the *S*-*R* line (green). Only the valence bands are shown for 0 ≤ *k*_*z*_ ≤ *π*. Cross section of the loop nodes are marked in circles. The Parameters are the same as in Fig. 3.

We find the two loops determined by Eqs (19) and (21) are linked, as shown in Fig. 4.

The system is a trivial insulator for 0 ≤ *t*_*z*_ < *t*_1_. One loop emerges for *t*_1_ < *t*_*z*_ < *t*_2_ [Fig. 3(b1)], which splits into two loops for *t*_2_ < *t*_*z*_ < *t*_3_, as shown in Fig. 3(d1). Correspondingly, drum-head surface states, which are partial flat band within the loop nodes, appear along the [100] surface [see Fig. 3(b3,c3 and d3)].

The emergence of the loop-nodal Fermi surface is understood in terms of the band inversion^20,40^, as shown in Fig. 4. The number of the loops are identical to the number of circles at the Fermi energy as in Fig. 4(a2–l2). When only one band is inverted along the Γ-*Z* line, a single loop node appears [Fig. 4(b1)]. When two bands are inverted along the Γ-*Z* line, two loop nodes appear [Fig. 4(d1)]. In the similar way, additional loops appear when additional bands are inverted along the *X*-*U* and *Y*-*T* lines [Fig. 4(f1)], and it is split into two loops [Fig. 4(h1)] as *t*_*z*_ increases. In the final process, a loop appears along the *S*-*R* line [Fig. 4(j1)], which splits into two loops [Fig. 4(l1)].

It has been argued^20,40^ that a new topological nature of loop-nodal semimetals becomes manifest when we plot the loop-nodal Fermi surfaces at the band crossing energies, where one is at the Fermi energy and the other is at *E* = −*m*_Loop_ in the occupied band. We show them in Fig. 4. Along the Γ−*Z* line, the other band crossing occurs at ±*m*_Loop_ with

22 $$|{k}_{z}|=\arccos \,[({m}_{{\rm{Loop}}}-m-2t)/{t}_{z}].$$

Along the *X*-*U* and *Y*-*T* lines, the band crossing occurs also at ±*m*_Loop_ with

23 $$|{k}_{z}|=\text{arccos}\,[\,-\,m/{t}_{z}].$$

Along the *S*-*R* line, the band crossing occurs also at ±*m*_Loop_ with

24 $$|{k}_{z}|=\arccos \,[(\,-\,m+2t)/{t}_{z}].$$

As a result, it is enough to plot the Fermi surfaces at *E* = 0 and *E* = −*m*_Loop_. The linking number *N* increases as *t*_*z*_ increases, where even the linking number *N* = 8 is realized as in Fig. 4(l1).

### 2D TI, TCI and SOTI

At this stage it is convenient to study the 2D models by setting *t*_*z*_ = *λ*_*z*_ = 0. It follows from (17) that the 2D topological phase boundaries are given by

25 $${(m+{\eta }_{x}{t}_{x}+{\eta }_{y}{t}_{y})}^{2}={m}_{{\rm{Loop}}}^{2}+{m}_{{\rm{SOTSM}}}^{2},$$

where *η*_*x*_ = ±1 and *η*_*y*_ = ±1. Depending on the way to introduce the mass parameters there are three phases, i.e., TIs, TCIs and SOTIs.

**Figure 5:**
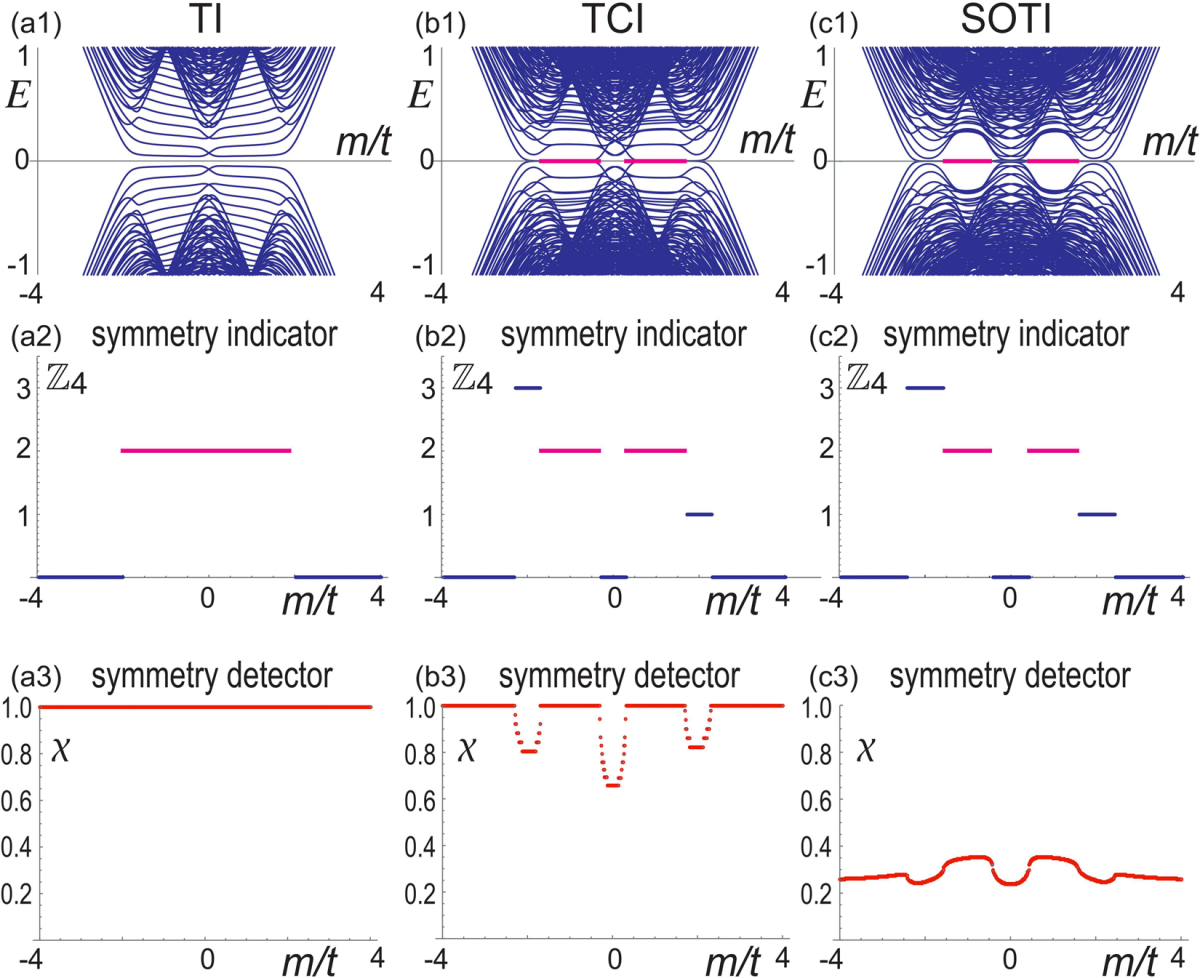
(**a1**–**c1**) Energy spectrum as a function of *m*/*t* for TI, TCI and SOTI phases. (**a2**–**c2**) Corresponding ${{\mathbb{Z}}}_{4}$ index. (**a3**–**c3**) Corresponding mirror-symmetry detector *χ*. It follows that *χ* = 1 for the TI and the insulating phase of the TCI, and that *χ* ≠ 1 for the SOTI since the mirror symmetry is broken.

The topological number is known to be the ${{\mathbb{Z}}}_{4}$ index protected by the inversion symmetry in three dimensions^20,41–43^. This is also the case in two dimensions. It is defined by

26 $${\kappa }_{1}\equiv \frac{1}{4}{\sum }_{K\in {\rm{TRIMs}}}({n}_{K}^{+}-{n}_{K}^{-}),$$

where ${n}_{K}^{\pm }$ is the number of occupied band with the parity ±. There is a relation^41–43^

27 $${{\rm{mod}}}_{2}{\kappa }_{1}=\nu ,$$

where *ν* is the ${{\mathbb{Z}}}_{2}$ index characterizing the time-reversal invariant TIs. We find from Fig. 5(c1) that *κ*_1_ = 0, 2 in the TI phase, which implies that it is trivial in the viewpoint of the time-reversal invariant topological insulators.

**Figure 6:**
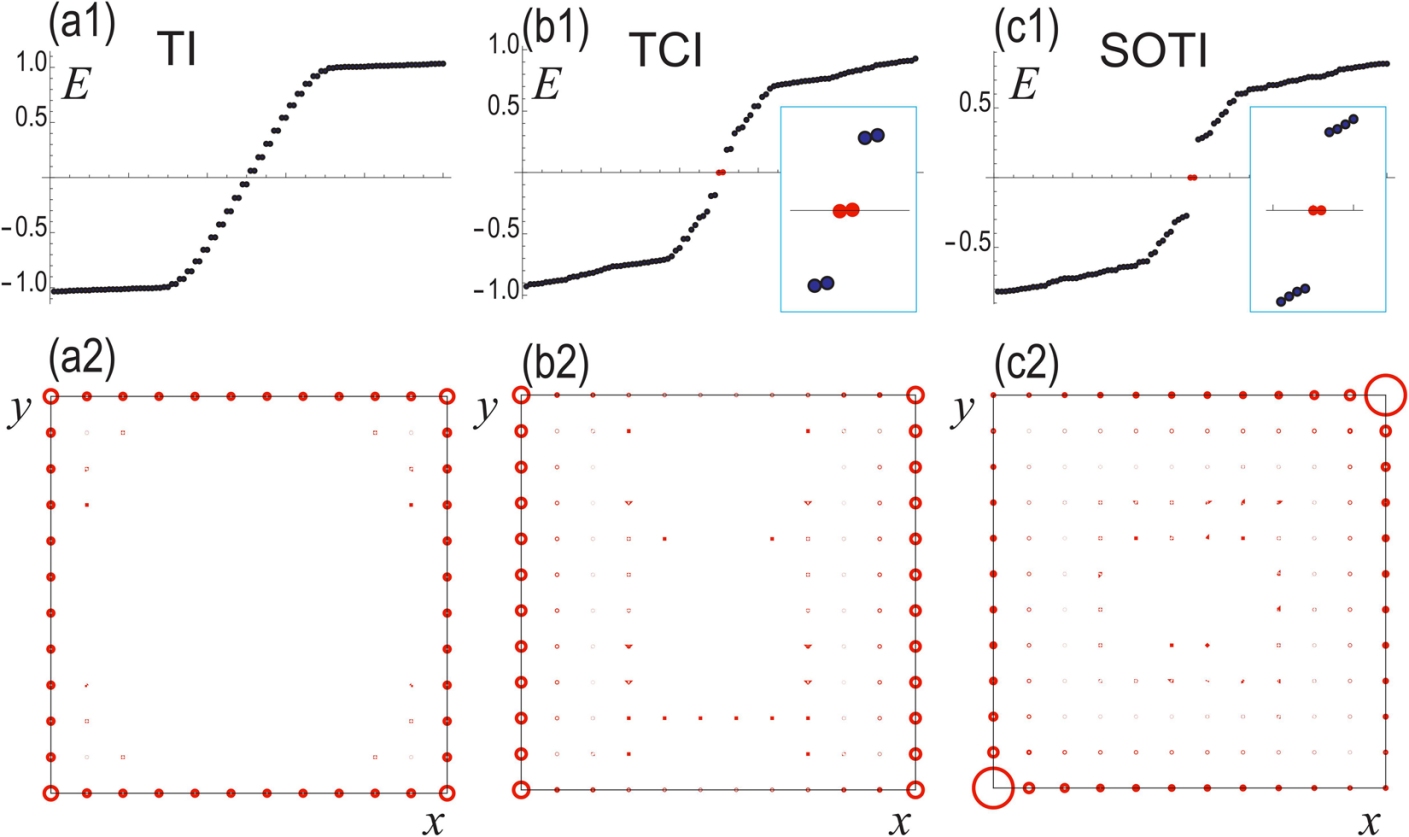
(**a1**–**c1**) Eigenvalues of the sample in a square geometry, where the insets show the zero-energy states in red. The vertical axis is the energy. (**a2**–**c2**) corresponding LDOS of the zero-energy states. The amplitude is represented by the radius of the circles. We have set *t*_*x*_ = *t*_*y*_ = *m* = *λ* = 1 and *m*_Loop_ = *m*_SOTSM_ = 0.3.

We show the LDOS for TI, TCI and SOTI in Fig. 6. (i) When *m*_Loop_ = *m*_SOTSM_ = 0 and |*m*| < 2*t*, the system is a TI with *κ*_1_ = 2, where topological edge states appear for all edges [See Fig. 6(a)]. We show the energy spectrum and the *Z*_4_ index in Fig. 5(a1,a2), respectively. The energy spectrum is two-fold degenerate since there is the symmetry $P\bar{T}={\mu }_{y}$ such that ${(P\bar{T})}^{-1}{H}_{0}(k)P\bar{T}={H}_{0}(k)$. Furthermore, there is the mirror symmetry *M*_*x*_ = *iτ*_*z*_*μ*_*z*_ such that ${M}_{x}^{-\,1}{H}_{{\rm{Loop}}}({k}_{x},{k}_{y}){M}_{x}={H}_{{\rm{Loop}}}(\,-\,{k}_{x},{k}_{y})$. (ii) When *m*_Loop_ ≠ 0 and *m*_SOTSM_ = 0, the system is a TCI, where topological edge states appear only for two edges [See Fig. 6(b)]. The energy spectrum and the *Z*_4_ index are shown in Fig. 5(b1,b2). The symmetry $P\bar{T}$ is broken for *m*_Loop_ ≠ 0 and the two-fold degeneracy is resolved. On the other hand, the mirror symmetry *M*_*x*_ remains preserved. (iii) Finally, when *m*_Loop_ ≠ 0 and *m*_SOTSM_ ≠ 0, the system is a SOTI, where two corner states emerge [See Fig. 6(c)]. The energy spectrum and the *Z*_4_ index are shown in Fig. 5(c1,c2). The mirror symmetry is broken in the SOTI phase. In TCI and SOTI phases, there are regions where *κ*_1_ = 1, 3. However, in this region, the system is semimetallic and the *κ*_1_ index has no meaning.

The *Z*_4_ index takes the same value for the TI, TCI and SOTI phases, and hence it cannot differentiate them. Indeed, because there is no band gap closing between them^44^, the symmetry indicator cannot change its value^43^. A natural question is whether there is another topological index to differentiate them. We propose the symmetry detector discriminating whether the symmetry is present or not.

The TI and TCI are differentiated whether the symmetry $P\bar{T}$ is present or not. The band is two-fold degenerate due to the symmetry $P\bar{T}$ in the TI phase, where we can define a topological index by

28 $$\zeta ={{\rm{Mod}}}_{4}\sum _{K\in {\rm{TRIMs}}}\,\frac{{\rm{Pf}}[w]}{\sqrt{{\rm{\det }}[w]}}$$

with

29 $${w}_{ij}=\langle {\psi }_{i}(\,-\,K)|P\bar{T}|{\psi }_{j}(K)\rangle ,$$

where *i* and *j* are the two-fold degenerated band index. It is only defined for the TI phase, where it gives the same result as *κ*_1_. On the other hand, it is ill-defined for the TCI and SOTI phases since there is no band degeneracy.

The TCI and SOTI are differentiated by the mirror-symmetry detector defined by

30 $$\chi \equiv {\chi }_{0}^{+}{\chi }_{\pi }^{+}{\chi }_{0}^{-}{\chi }_{\pi }^{-},$$

where

31 $${\chi }_{\alpha }^{\pm }\equiv \frac{-i}{2\pi }{{\int }_{0}^{2\pi }\langle \psi |{M}_{x}|\psi \rangle d{k}_{y}|}_{{k}_{x}=\alpha }$$

is the mirror symmetry indicator^36^ along the axis *k*_*x*_ = *α* with *α* = 0,*π*, and ± indicates the band index under the Fermi energy. It is *χ* = 1 when there is the mirror symmetry. On the other hand, it is *χ* ≠ 1 when there is no mirror symmetry since $|\psi \rangle$ is not the eigenstate of the mirror operator. In addition, it is *χ* ≠ 1 when the system is metallic since $\langle \psi |{M}_{x}|\psi \rangle$ changes its value at band gap closing points. See Fig. 5(a3–c3). In Fig. 5(a3), we find always *χ* = 1 since the mirror symmetry is preserved, where we cannot differentiate the topological and trivial phases. On the other hand, in Fig. 5(b3), there are regions with *χ* ≠ 1 where the system is metallic. Finally, we find *χ* ≠ 1 in Fig. 5(c3) since the mirror symmetry is broken.

### SOTSM

A 3D SOTSM is constructed by considering *k*_*z*_ dependent mass term in the 2D SOTI model^10,12,13^. We set *t*_*z*_ ≠ 0, while keeping *λ*_*z*_ = 0 in the 2D SOTI model. The properties of the SOTSM are derived by the sliced Hamiltonian *H*(*k*_*z*_) along the *k*_*z*_ axis, which gives a 2D SOTI model with *k*_*z*_ dependent mass term *M*(*k*_*z*_). The bulk band gap closes at

32 $${M}^{2}({k}_{z})={m}_{{\rm{Loop}}}^{2}+{m}_{{\rm{SOTSM}}}^{2}.$$

On the other hand, there emerge hinge-arc states connecting the two gap closing points. Accordingly, the topological corner states in the 2D SOTI model evolves into hinge-states, whose dispersion forms flat bands as shown in Fig. 2(c4).

### Magnetic control of hinges in SOTI

Hinge states are analogous to edge states in two-dimensional topological insulators. Without applying external field, spin currents flow. On the other hand, once electric field is applied, charge current carrying a quantized conductance flows. We show that the current is controlled by the direction of magnetization as in the case of topological edge states.

With the inclusion of the *H*_SO_, the system turns into a SOTI, which has topological hinge states. We study the effects of the Zeeman term, where the Hamiltonian is described by *H*_SOTI_ together with the Zeeman term

33 $${H}_{{\rm{Z}}}={B}_{x}{\sigma }_{x}+{B}_{y}{\sigma }_{y}+{B}_{z}{\sigma }_{z},$$

which will be introduced by magnetic impurities, magnetic proximity effects or applying magnetic field.

**Figure 7:**
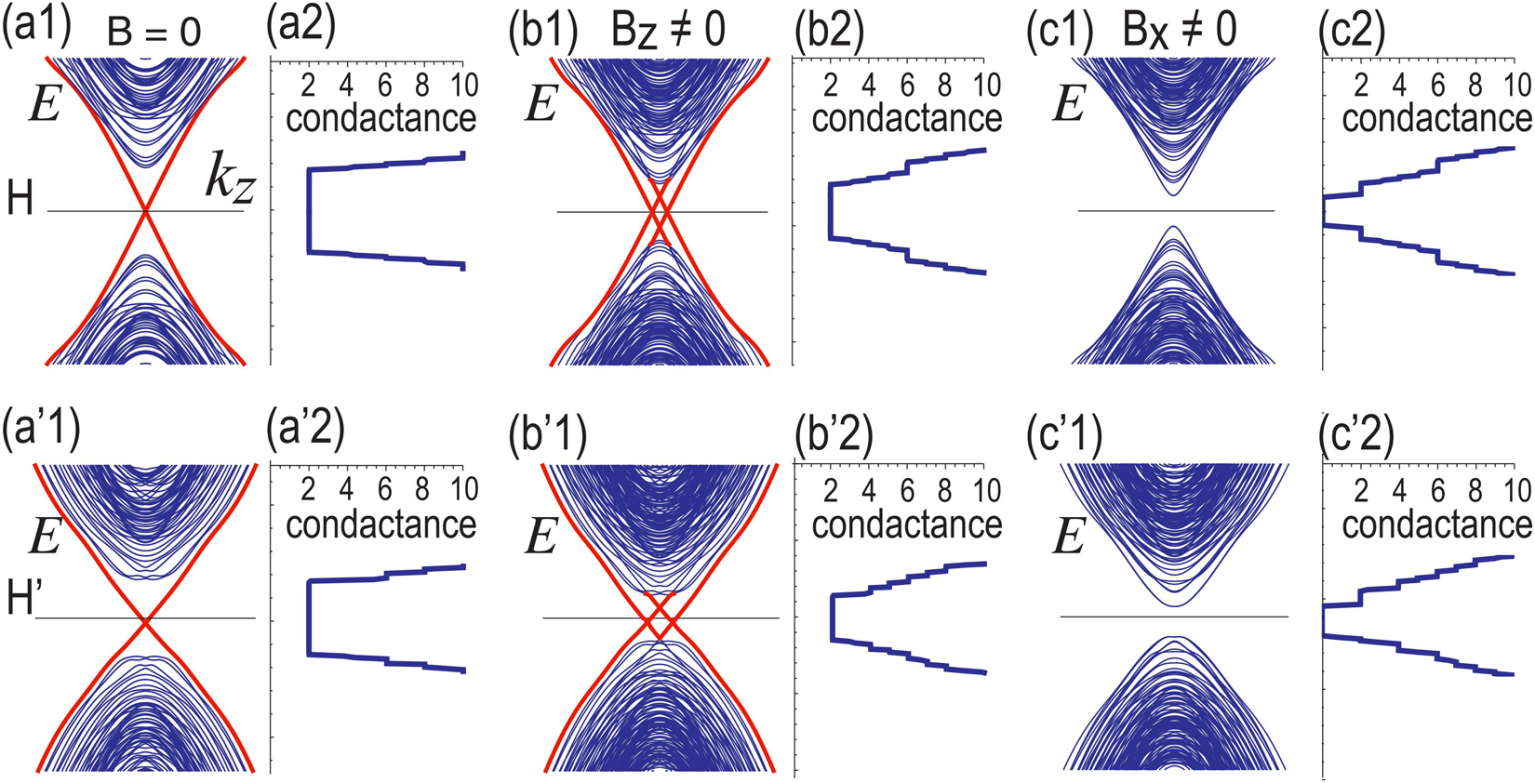
Band structures for hinge states (**a1**) without magnetic field, (**b1**) with magnetic field along the *z* direction and (**c1**) with magnetic field along the *x* direction for the chiral-symmetric Hamiltonian *H*_SOTI_. Hinge states are depicted in red. (**a2**–**c2**) Corresponding ones for the chiral-nonsymmetric Hamiltonian ${H}_{{\rm{SOTI}}}^{^{\prime} }$. (**a2**–**c2**) and (**a′2**–**c′2**) The conductance is quantized proportional to the number of bands in various cases.

We show the hinge states in the absence and the presence of magnetization in Fig. 7. Helical hinge states appear in its absence [see Fig. 7(a1)]. They are shifted in the presence of the *B*_*z*_ term [see Fig. 7(b1)]. On the other hand, they are gapped out when the *B*_*x*_ or *B*_*y*_ term exists [see Fig. 7(c1)].

For comparison, we also show the hinge states calculated from the chiral-nonsymmetric Hamiltonian ${H}_{{\rm{SOTI}}}^{^{\prime} }$ [see Fig. 7(a2–c2)]. The band structure is almost symmetric with respect to the Fermi energy.

By taking into the fact that the *σ*_*z*_ is a good quantum number, the low energy theory of the hinge states is well described by

34 $$H=\hslash {v}_{{\rm{F}}}{k}_{z}{\sigma }_{z}.$$

In the presence of the external magnetic field, it is modified as

35 $$H=\hslash {v}_{{\rm{F}}}{k}_{z}{\sigma }_{z}+{B}_{x}{\sigma }_{x}+{B}_{y}{\sigma }_{y}+{B}_{z}{\sigma }_{z},$$

which is easily diagonalized to be

36 $$E=\pm \,\sqrt{{(\hslash {v}_{{\rm{F}}}{k}_{z}+{B}_{z})}^{2}+{B}_{x}^{2}+{B}_{y}^{2}}.$$

It well reproduces the results based on the tight binding model shown in Fig. 7.

One of the intrinsic features of a topological hinge state is that it conveys a quantized conductance in the unit of *e*^2^/*h*. We have calculated the conductance of the hinge states in Fig. 7 based on the Landauer formalism^45–51^. In terms of single-particle Green’s functions, the conductance *σ*(*E*) at the energy *E* is given by^45,51^

37 $$\sigma (E)=({e}^{2}/h){\rm{Tr}}[{{\rm{\Gamma }}}_{{\rm{L}}}(E){G}_{{\rm{D}}}^{\dagger }(E){{\rm{\Gamma }}}_{{\rm{R}}}(E){G}_{{\rm{D}}}(E)],$$

where ${{\rm{\Gamma }}}_{R(L)}(E)=i[{{\rm{\Sigma }}}_{R(L)}(E)-{{\rm{\Sigma }}}_{R(L)}^{\dagger }(E)]$ with the self-energies Σ_L_(*E*) and Σ_R_(*E*), and

38 $${G}_{{\rm{D}}}(E)={[E-{H}_{{\rm{D}}}-{\Sigma }_{{\rm{L}}}(E)-{\Sigma }_{{\rm{R}}}(E)]}^{-1},$$

with the Hamiltonian *H*_D_ for the device region. The self energies Σ_L_(*E*) and Σ_R_(*E*) are numerically obtained by using the recursive method^45–51^.

The conductance is quantized, which is proportional to the number of bands. When there is no magnetization or the magnetization is along the *z* axis, the conductance is 2 since there are two topological hinges. On the other hand, once there is in-plane magnetization, the conductance is switched off since the hinge states are gapped. It is a giant magnetic resistor^35^, where the conductance is controlled by the magnetization direction.

## Conclusion

We have studied chiral-symmetric models to describe SOTIs and loop-nodal semimetals in transition metal dichalcogenides. The Hamiltonian is analytically diagonalized due to the chiral symmetry. We have obtained analytic formulas for various phases including loop-nodal semimetals, 2D SOTIs, 3D SOTSMs and 3D SOTIs. We have proposed the symmetry detector discriminating whether the symmetry is present or not. It can differentiate topological phases to which the symmetry indicator yields an identical value. Furthermore, we have proposed a topological device, where the conductance is switched by the direction of magnetization. Our results will open a way to topological devices based on transition metal dichalcogenides.
